# 

**Published:** 1889-08-24

**Authors:** 


SATURDAY, AUGUST 24th, 1889.

**A Doctor's Vision** - - - 319
**Words of Consolation**—The Lessons Taught by Suffering 320
**On Some Points Connected with Cholera.** II.—Some Theories Examined - - - 321
**Within the Wards.**—A Pasteur Case - - - 322
**The Doctor's Pulpit.**—In Nature's School - - - 323
**Nightmare.** By SIR WILLIAM MOORE, K.C.I.E. - - - 324
**Torturing and Starving Fish.** By J. LAURENCE-HAMILTON, M.R.C.S. - - - 325
**Notes and News** - - - 326
**Everybody's Page** - - - 328
**Annotations.**—What Should the Deaf Do?—Troubled Waters—A Court of Criminal Appeal - - - 329
**The Plans and Purposes of the Johns Hopkins Hospital.** II.—Cardinal Principles. By JOHN S. BILLINGS, M.D., Surgeon, U.S. Army - - - 330
**Her Heart's Mistake.** By E. D. CUMMING. Chap. xix. - 331
**Wanted, More Howards** - - - 332
**Amusements and Relaxation** - - - 334
**Scraps and Gleanings** - - - 334

EXTRA SUPPLEMENT—THE NURSING MIRROR.

**En Passant.**—The Doll Competition—The Midwives' Institute—Reminiscences of Dr. Rogers—Causes of Death—Another Absent Nurse—Diphtheria—Deaconesses at Utrecht—Lady Dentists - - - lxxix

**Winter Amusements and Remunerative Work.**—An Exhibition of Doll Nurses - - - lxxx

**Notes from Australia** - - - lxxx

**Physiology for Nurses.** No. 6.—On the Skeleton - lxxxi

**The Nurse's Bookshelf.**—Another Manual - - lxxxii

**Everybody's Opinion.**—Clean Nails - - - lxxxii

**Presentation** - - - lxxxii

**Vacancies—Notes and Queries** - - - lxxxii

